# The Molecular Recognition of Lurasidone by Human Serum Albumin: A Combined Experimental and Computational Approach

**DOI:** 10.3390/molecules30071420

**Published:** 2025-03-22

**Authors:** Nevena Živković, Emina Mrkalić, Ratomir Jelić, Jovica Tomović, Jadranka Odović, Marina Ćendić Serafinović, Miroslav Sovrlić

**Affiliations:** 1 Department of Pharmacy, Faculty of Medical Sciences, University of Kragujevac, Svetozara Markovića 69, 34000 Kragujevac, Serbia; nensyprodanovic@yahoo.com (N.Ž.); rjelic@kg.ac.rs (R.J.); sofke-ph@hotmail.com (M.S.); 2 Department of Science, Institute for Information Technologies, University of Kragujevac, Jovana Cvijića bb, 34000 Kragujevac, Serbia; 3 Faculty of Pharmacy, University of Belgrade, Vojvode Stepe 450, 11221 Belgrade, Serbia; jodovic@pharmacy.bg.ac.rs; 4 Department of Chemistry, Faculty of Science, University of Kragujevac, Radoja Domanovića 12, 34000 Kragujevac, Serbia; marina.cendic@pmf.kg.ac.rs

**Keywords:** lurasidone, albumin, protein binding, circular dichroism, fluorescence spectroscopy, molecular modeling

## Abstract

Lurasidone (LUR) is an antipsychotic drug whose interaction with human serum albumin (HSA) plays a crucial role in its pharmacokinetic and pharmacodynamic properties. A thorough understanding of LUR’s binding mechanism to HSA is crucial for predicting its transport, distribution, and potential drug interactions. Methods: The interaction between LUR and HSA was investigated using fluorescence and circular dichroism (CD) spectroscopy, followed by molecular docking simulations. Binding characteristics were analyzed through quenching mechanisms, thermodynamic parameters, and competitive site marker experiments. Results: This study revealed a systematic decrease in HSA fluorescence intensity with increasing LUR concentration, indicating a static quenching mechanism driven by non-fluorescent complex formation. Binding constants suggest enhanced complex stability at higher temperatures, with thermodynamic analysis confirming an endothermic, hydrophobic interaction. Competitive site marker assays and synchronous fluorescence spectra confirmed that LUR primarily binds to site I (subdomain IIA) near tryptophan residues. Conformational changes in HSA, observed as a decrease in α-helix content, further demonstrate the structural impact of LUR binding. Conclusions: These findings offer key insights into the molecular interactions between LUR and HSA, enhancing our understanding of LUR’s pharmacokinetics and its potential interactions with other drugs. Understanding these binding characteristics can aid in optimizing LUR’s clinical application and predicting possible interactions with other biomolecules.

## 1. Introduction

Molecular recognition is essential for physiological processes, including signal transduction, immune response, and enzymatic activity. Highly efficient and specific recognition and binding, prerequisites for enzyme-catalyzed reactions, play key roles in initiating and regulating a metabolic network consisting of several thousand chemical reactions occurring in parallel [1]. The drug–HSA complex serves as a reservoir, gradually releasing the drug as the free drug concentration in plasma decreases. In this way, a dynamic balance between the free and bound fraction of the drug is achieved [2]. HSA is the most abundant protein in the extracellular fluid, constituting approximately 60% of the mass of all proteins in plasma [3]. The most important roles of HSA are the maintenance of colloid-osmotic pressure and the transport of endogenous and exogenous ligands. HSA has an antioxidant effect so that it protects the drug from oxidation and also affects the pharmacokinetic and pharmacodynamic properties of the drug [4]. According to the analyses of the crystal structure of HSA published so far, there are three main binding sites with strong affinity for a large number of ligands. Two binding sites are located in subdomains IIA and IIIA (Sudlow site I and Sudlow site II) and represent the main drug binding sites [5]. Recent research has shown the existence of another important binding site to which heme binds, but also many other ligands (antitumor drugs, anticoagulants, non-steroidal anti-inflammatory drugs). This binding site is located in subdomain IB [6].

Fluorescence spectroscopy of proteins sees extremely wide application to obtain structural and dynamic data related to the immediate environment of fluorophores in large molecules. The internal fluorescence of HSA originates from three main amino acid residues, namely: tryptophan (TRP), tyrosine (TYR) and phenylalanine (PHE). Protein fluorescence is generally observed at wavelengths of 280 nm or longer (most often at 295 nm). At 295 nm, fluorescence originates mostly from Trp, while at 280 nm, fluorescence originates from a combination of Trp, Tyr, and Phe residues. The wavelengths of maximum absorption and emission are important characteristics for defining each fluorophore [7,8].

Lurasidone (LUR) is atypical antipsychotic of the benzisothiazole class (3aR,4S,7R,7aS)-2-[(1R,2R)-2-[4-(1,2-benzisothiazol-3-yl) piperazin-1-ylmethyl] cyclohexylmethyl] hexahydro-4, 7-methano-2H-isoindole-1,3-dione hydrochloride) of chemicals [9]. Similar to many other second-generation antipsychotics, lurasidone fully blocks dopamine D_2_ and serotonin 5-HT_2A_ receptors, while acting as a partial activator of 5-HT_1A_ receptors. This partial agonism at 5-HT_1A_ receptors is a characteristic shared with some older antipsychotic drugs, although not all of them. With its receptor profile, LUR offers several advantages—reducing negative symptoms, enhancing cognition and circadian rhythm regulation, improving depressive symptoms, and minimizing drowsiness and somnolence—due to its moderate affinity as an α-2A adrenergic agonist, all without causing sedation [10]. LUR is a medication used in the treatment of schizophrenia and bipolar depression [11]. Schizophrenia is one of the most debilitating psychiatric disorders and affects about 1% of the population worldwide. When considering both conditions, the use of lurasidone seems to have a minimal impact on body weight and a low likelihood of causing significant changes in glucose, lipids, or ECG parameters. Lurasidone’s combination of efficacy in schizophrenia and bipolar depression with minimal metabolic disturbance and little effect on movement disorders and prolactin represents a potentially important clinical advance. LUR is approved up to 160 mg/day for schizophrenia, though higher dosages have only been studied for safety, not efficacy; it should be taken with food to increase absorption and is typically administered once daily to enhance compliance via regimen simplicity. It is approved for use in adolescents with schizophrenia, expanding its applicability to a broader patient population. By effectively managing symptoms with fewer side effects, lurasidone can significantly improve the quality of life for patients, allowing for better social and occupational functioning [10,12]. LUR displays a high degree of binding to human plasma albumin and alpha-1-glycoprotein (≥99%). The mean fraction of LUR distributed in red blood cells was approximately 12% in vivo in healthy subjects [13]. The potential for protein-based clinical drug-–drug interactions appeared to be minimal as no displacement of LUR or co-incubated drugs (biperiden, flunitrazepam, haloperidol, or diazepam) was observed from serum proteins in vitro. Similarly, the potential for clinical drug–drug interactions mediated by the inhibition or induction of CYP activity by LUR is low, since in vitro studies using human tissue preparations suggest that, at clinically relevant concentrations, LUR does not inhibit or induce CYP enzyme activity [14].

The purpose of this study is to comprehensively investigate the binding features between and HSA, aiming to gain a detailed understanding of lurasidone’s affinity for HSA. The objectives include elucidating the driving forces behind HSA–LUR complex formation, identifying the specific amino acid residues contributing to the conformation of the complex, and assessing the impact on the conformation of HSA. Importantly, while lurasidone’s pharmacokinetics and safety profile have been characterized clinically, there is a notable lack of molecular-level insights into its binding behavior with plasma proteins. To the best of our knowledge, this is the first comprehensive study combining experimental spectroscopic techniques with molecular docking simulations to investigate the molecular recognition of lurasidone by HSA. This integrated approach aims to provide new theoretical insights into LUR’s in vivo transport and distribution behavior, as well as its potential binding mechanisms and pharmacokinetics, which are essential for optimizing its therapeutic efficacy and predicting drug interactions.

## 2. Results and Discussion

### 2.1. Characterisation of LUR Binding to HSA

**Figure 1:**
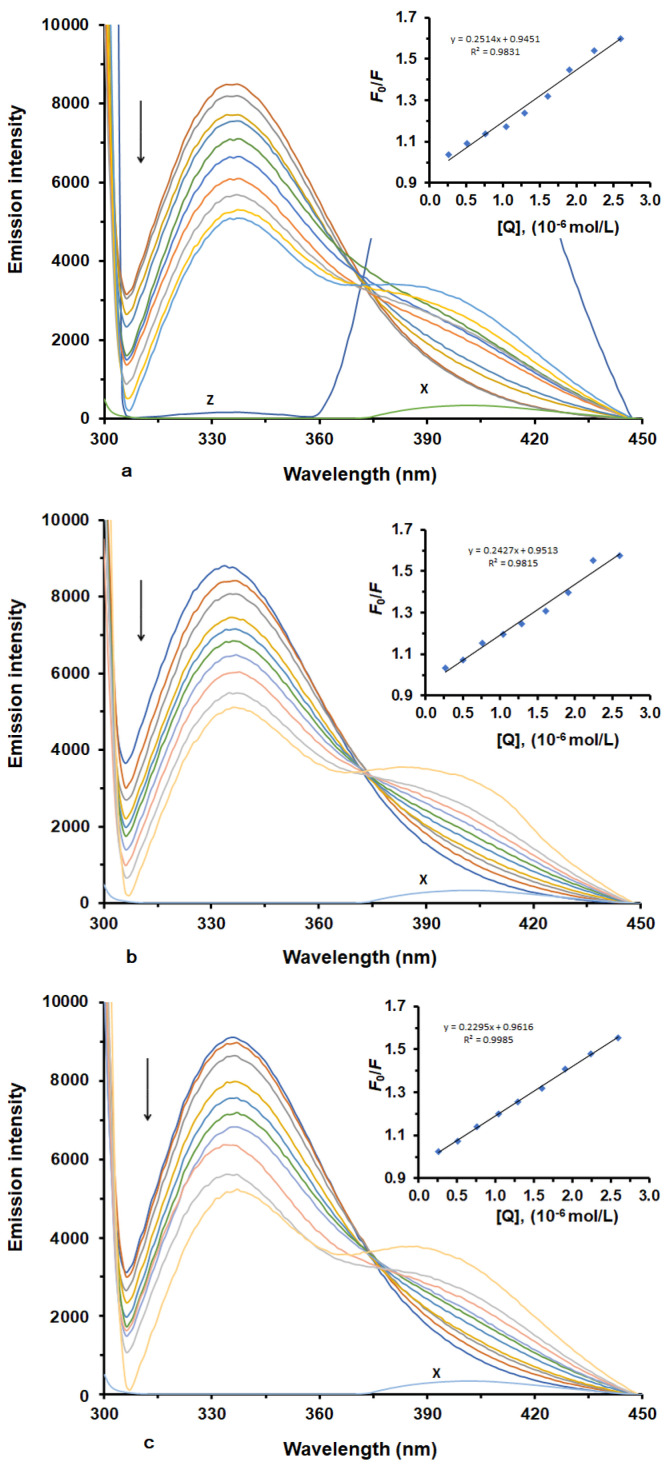
Fluorescence emission spectra and corresponding Stern–Volmer plots (inset) of HSA in the presence of various concentrations of LUR at three different temperatures ((**a**) T = 298 K; (**b**) T = 303 K; (**c**) T = 308 K). X represents LUR only. Z represents HSA-LUR (1:4).

To analyze LUR-HSA binding, we recorded fluorescence emission spectra of HSA under two conditions: with and without LUR. The fluorescence quenching spectra of HSA in the presence of different concentrations of LUR at three different temperatures are illustrated in (Figure 1a–c). HSA exhibits a robust fluorescence emission peak at 337 nm when excited at 280 nm (Figure 1). This emission arises from tryptophan (Trp) and tyrosine (Tyr) residues in HSA [15,16]. Upon adding varying concentrations of LUR to the HSA solution, there was a systematic decrease in HSA’s fluorescence intensity with an increasing LUR concentration. This result indicates the existence of an interaction between LUR and HSA, causing the quenching of HSA’s intrinsic fluorescence.

Fluorescence quenching can be categorized into two main types—dynamic and static quenching—which result from diffusion or the formation of ground-state complexes, respectively [17]. Typically, these two quenching mechanisms can be differentiated based on their distinct dependencies on quenching constants, viscosity, or, preferably, lifetime measurements [18].

In our study, to ascertain the quenching mechanism involved in the interaction between HSA and LUR, we calculated fluorescence quenching constants at various temperatures (298, 303, and 308 K) using the Stern–Volmer equation [19]. Figure 1 displays Stern–Volmer plots (inset), depicting F_0_/F as a function of [LUR] at three distinct temperatures, while Table 1 shows the values calculated for *K*_SV_ and *K*_q_. As seen, the plots of F_0_/F versus [LUR], ranging from 0 to 3.2 × 10^−6^ M, are linear. This result implies that a single quenching mechanism was in operation at these concentrations, and this mechanism could be either static or dynamic [20,21]. Dynamic quenching is marked by an increase in the quenching constant with a rising temperature [22], while a decreasing quenching constant with a rising temperature implies there is a static quenching mechanism. In our experiment, *K*_SV_ values decreased with an increase in temperature, indicating the static quenching mechanism occurred through an interaction between LUR and HSA. Further, the Stern–Volmer quenching constants (*K*_q_) are greater than 2.0 × 10^10^ M^−1^ s^−1^ (the maximum characteristic value of *K*sv for the dynamic quenching mechanism), which usually indicates that the quenching mechanism is static and likely involves the formation of a non-fluorescent ground-state complex between the ligand and the protein.

These findings align with previous studies on the static quenching mechanism observed in the binding of various antipsychotic drugs with serum albumins, such as risperidone and aripiprazole, which also demonstrated temperature-dependent decreases in quenching constants and the formation of stable drug–protein complexes [23,24]. Compared to these, lurasidone exhibits similar quenching behaviour, indicating the existence of a common mode of interaction among atypical antipsychotics with HSA. This comparative insight not only confirms the static quenching mechanism of LUR but also reinforces the relevance of temperature in modulating drug–protein interactions, which can critically affect pharmacokinetics and bioavailability. Furthermore, understanding such binding dynamics is essential for optimizing drug dosing regimens and predicting drug–drug interactions in polypharmacy settings, particularly in psychiatric treatments where multiple drugs are often co-administered [25,26]. Consequently, these results contribute valuable knowledge to the growing body of research focused on the molecular recognition of central nervous system drugs by plasma proteins, with potential implications for therapeutic monitoring and individualized medicine.

### 2.2. Determination of Binding Constant and Binding Stoichiometry

**Figure 2:**
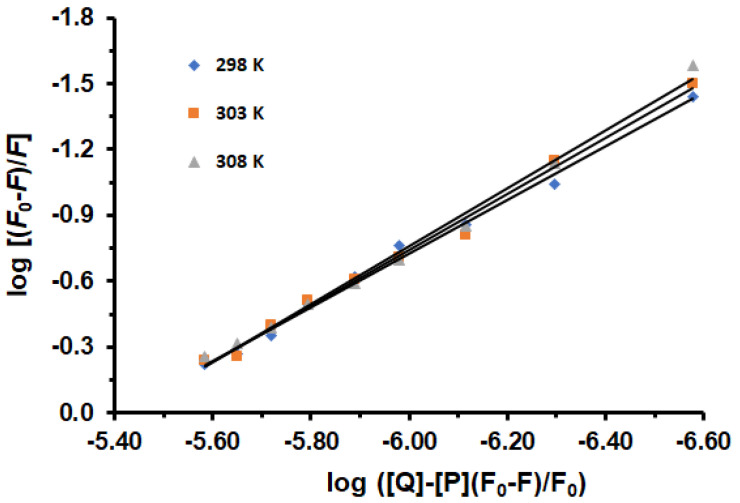
Logarithmic plots of HSA fluorescence quenching caused by LUR at 298, 303, and 308 K: [HSA] = 1.6 × 10^−6^ M, [LUR] = 0–3.2 × 10^−6^ M.

In the case of static quenching, the binding constant (*K*a) and the binding sites (n) for the reaction between LUR and HSA can be obtained from Equation (3). Since the equilibrium quencher concentration (LUR) was not known, the total LUR concentration, which was known from the experiment, was used in the calculation of *K*a and n. Figure 2 displays the plots of log (F_0_ − F)/F against log([Q] − [P](F_0_ − F/F_0_) for the HSA-LUR system in the absence and presence of increasing concentrations of LUR.

The *K*_a_ and n values are listed in Table 2. It is evident that as temperatures increase, the *K*_a_ values also increase, lead to improved stability of the HSA-LUR complex. The results indicate that the LUR-HSA interaction decreases the free drug concentration in plasma, potentially reducing drug efficacy. The values for the number of binding sites suggest that HSA and LUR interact at a molar ratio of 1:1.22, which equates to approximately 1 binding site for LUR on HSA. Through the experiment, the main site was observed. This was also shown by docking. However, additional binding sites are apparently observed, which requires further research. Also, a slight increase in the number of binding sites with an increasing temperature possibly indicates changes in experimental conditions or the presence of different protein conformations.

The most prominent interactions between drugs and proteins may be explained through thermodynamic parameters derived from the temperature dependence of binding constants. The nature of the interaction involves a combination of various weak forces. Typically, the signs and values of thermodynamic parameters, specifically the enthalpy change (Δ*H*^0^), entropy change (Δ*S*^0^), and Gibbs free energy change (Δ*G*^0^), are indicative of the primary forces governing the binding process. Drawing insights from the analysis of thermodynamic parameters, when Δ*H*^0^ is greater than 0 and Δ*S*^0^ is greater than 0, it suggests the presence of a hydrophobic interaction. Conversely, when Δ*H*^0^ is less than 0 and Δ*S*^0^ is less than 0, it points toward the formation of van der Waals forces or hydrogen bonds. Lastly, when Δ*H*^0^ is approximately 0 and Δ*S*^0^ is greater than 0, it hints at the involvement of an electrostatic force [27]. In this investigation, experiments were conducted within the temperature range of 298–308 K, a range where HSA experiences no substantial structural alterations [28].

**Figure 3:**
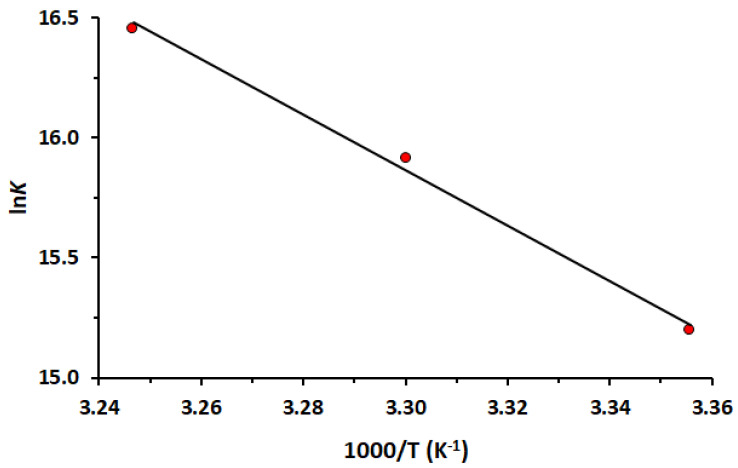
The van’t Hoff plot of the interaction between HSA and LUR at 298, 303 and 308 K.

Figure 3 shows the good linear dependence obtained by plotting. The Δ*H*^0^ and Δ*S*^0^ are obtained (Table 2) from the slope and intercept of the van’t Hoff curve, respectively. The positive values of Δ*H*^0^ and Δ*S*^0^ indicate hydrophobic interactions are the most important forces between HSA and LUR, while negative values of Δ*G*^0^ suggest that the binding process is spontaneous. A positive value of Δ*H*^0^ suggests that the interaction between the drug and HSA is endothermic. This means that the enthalpy contribution to the binding Gibbs energy is unfavorable, opposing binding. The binding occurs because the binding entropy overcomes the binding enthalpy. A positive value of Δ*S*^0^ implies that the system becomes more disordered during the interaction.

### 2.3. Site-Selective Binding of LUR to HSA

Determining the binding sites of a drug on HSA is crucial in understanding its interaction with biological molecules. Various experimental techniques, such as fluorescence spectroscopy and displacement assays, are employed to pinpoint these sites accurately [29]. These methods involve using specific probes or markers for known binding sites on HSA, allowing researchers to study ligand interactions comprehensively.

**Figure 4:**
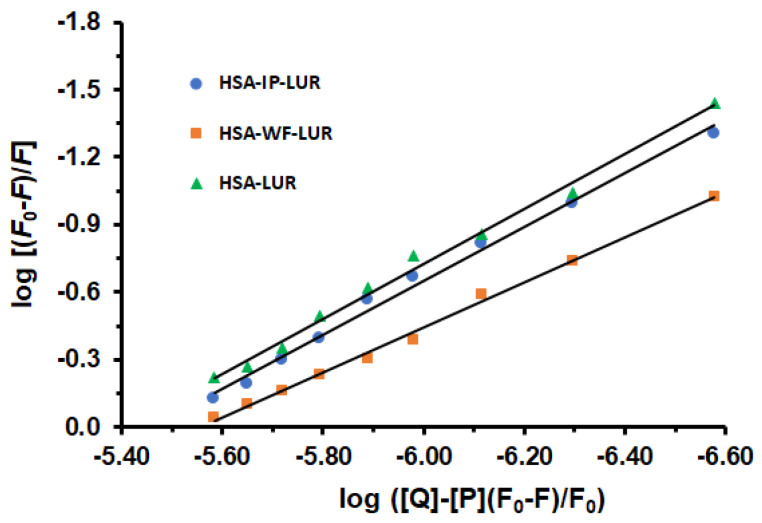
The effects of warfarin and ibuprofen on the fluorescence intensity of the HSA-LUR complex.

The primary regions where drugs bind to HSA are situated within the hydrophobic cavities in subdomains IIA and IIIA, identified as sites I and II, respectively [30]. To identify the LUR binding site in HSA, displacement experiments were conducted, using WF as a marker for site I and IP for site II. In this study, a solution containing equal concentrations of the site marker and HSA was initially prepared, as previously reported [31,32]. After a 30 min incubation at 298 K, increasing amounts of LUR were added. Binding constants (K_a_), and the number of binding sites (n) in the presence of site-specific markers (IP and WF), were calculated using Equation (3). Figure 4) displays the plots of log (F_0_ − F)/F against log([Q] − [P](F − F_0_)/F_0_) for the HSA-LUR, HSA-IP-LUR, and HSA-WF-LUR systems in the absence and presence of increasing concentrations of LUR.

The obtained values of binding constants of ternary systems HSA-IP-LUR and HSA-WF-LUR are *K*_a_ = 3.55 × 10^6^ M^−1^ and *K*_a_ = 3.47 × 10^5^ M^−1^, respectively. Comparing binding constants (*K*_a_) of the binary HSA-LUR system and ternary systems, it is evident that the presence of WF has a greater effect on the interactions between LUR and HSA than IP. Accordingly, the interaction between HSA and LUR was reduced in the presence of WF by 91.28% and fell in the presence of IP by 10.80%. The number of binding sites (n) on the binary HSA-LUR system was n = 1.22, while in ternary HSA-IP-LUR and HSA-WF-LUR systems this number was decreased to 1.19 and 0.99, respectively. Accordingly, these results indicate that the binding site of LUR is mainly located within site I (subdomain IIA) of HSA.

### 2.4. Conformational Changes in HSA Induced by the LUR

To explore the impact of LUR on HSA conformational changes upon binding to HSA, UV-vis spectroscopy, circular dichroism, and synchronous fluorescence spectroscopy were employed.

#### 2.4.1. UV-Vis Spectroscopy

**Figure 5:**
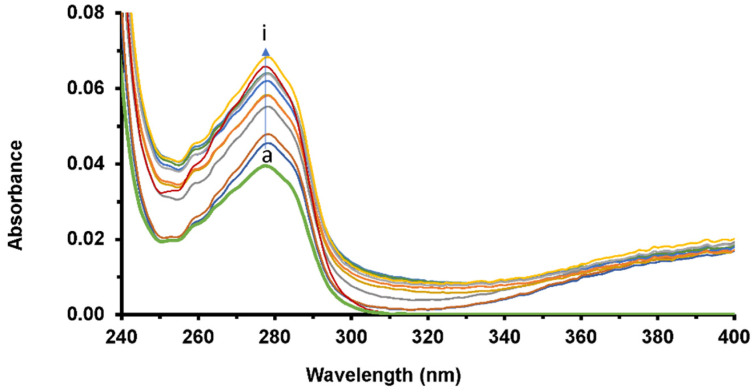
The absorption spectrum of HSA in the presence of increasing concentrations of LUR: [HSA] = 1.6 × 10^−6^ M, [LUR] = 0–3.5 × 10^−6^ M (a–i).

Absorption spectroscopy proves highly practical in studying systems featuring complex formation, which induces alterations in protein conformation. To investigate this phenomenon, UV-vis absorption spectra HSA were measured in the presence of increasing concentrations of LUR. These measurements were taken relative to a reference solution containing the drug at the appropriate concentration, facilitating the recording of spectra (Figure 5).

The green spectrum represents the HSA spectrum in the absence of LUR. In the spectra of free HSA, there is an absorption maximum at 278 nm, originating from the p–p* transition of aromatic amino acid residues (Trp, Tyr, and Phe) in HSA [33]. If there are no changes in the absorption spectra, this indicates a dynamic quenching effect that only affects excited states of the fluorophore. On the contrary, when fluorophores form a ground-state complex, it can be explained by the mechanism of static quenching, which can be observed based on changes in the absorption spectra. Based on the spectra obtained, the intensity of the peak at 278 nm increases with an increasing concentration of LUR. The spectra of HSA, in the absence and presence of LUR, showed that the interaction between LUR and HSA was achieved through a static process.

#### 2.4.2. CD Spectral Measurements

**Figure 6:**
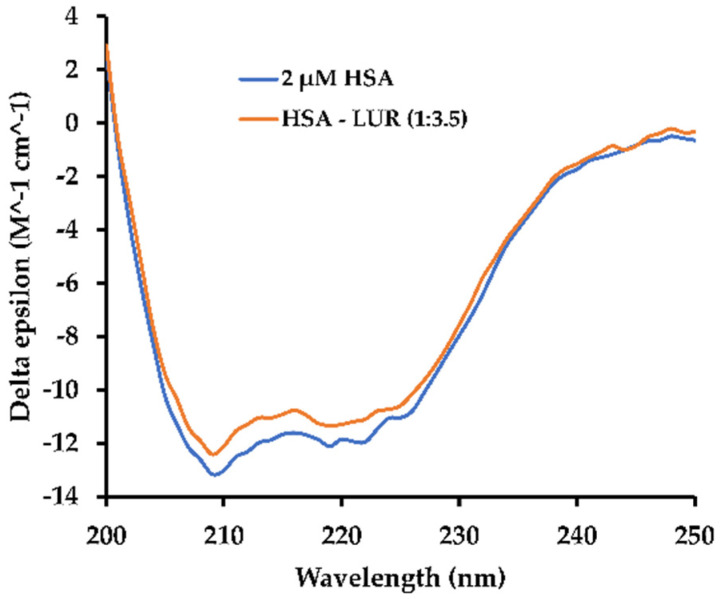
CD spectra of HSA in the absence and the presence of LUR: HSA:LUR = 1:3.5.

CD measurement serves as a potent method with which to examine the secondary structure of proteins. Typically, the CD spectrum in the far UV region, ranging from 200 to 250 nm, serves as a representative indicator of the protein’s secondary structure. Proteins rich in α-helices display a CD spectrum with two prominent negative absorption bands centred at 208 nm and 222 nm. In contrast, proteins abundant in β-sheets exhibit a single negative absorption band around 218 nm [34]. During the binding of ligands with proteins, intermolecular interaction forces can influence the microenvironment around the binding site, thereby leading to alterations in the protein’s secondary structure. Therefore, understanding the CD characteristics of HSA in the presence or absence of LUR is essential (Figure 6).

As is shown in Figure 6, a negative absorption band is observed near 222 nm in the HSA solution, corresponding to the n → π* transition in the α-helix structure [35]. Utilizing the BeStSel program [36], the α-helix content within the HSA secondary structure can be determined. Additionally, the α-helix content decreased from 68.7% in free HSA to 67.1% in the HSA-LUR system, suggesting a negligible conformational change in HSA upon LUR binding. It can be seen easily that alpha-helix amount slightly decreases, falling by 1–2%.

#### 2.4.3. Synchronous Fluorescence Spectral Measurements

**Figure 7:**
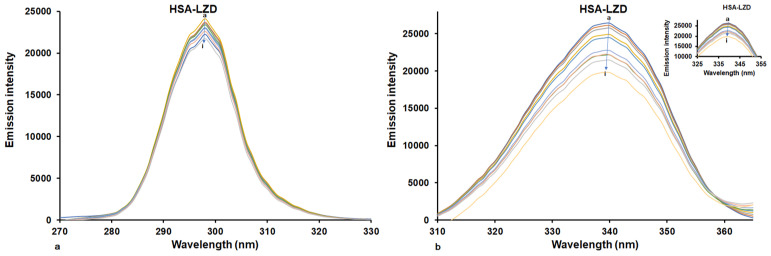
Synchronous fluorescence spectra of HSA in the absence and presence of LUR at 298 K: (**a**) Δλ values were fixed at 15 nm; (**b**) Δλ values were fixed at 60 nm. [HSA] = 1.6 × 10^−6^ M, [LUR] = 0–3.2 × 10^−6^ M (a–i).

Synchronous fluorescence spectroscopy is commonly employed to analyze alterations in the microenvironment surrounding specific amino acid residues upon ligand binding. The presence of exogenous substances may lead to the formation of complexes with proteins, causing conformational changes around the microenvironment of the chromophore. This method offers excellent selectivity, high sensitivity, and minimal interference. By setting the interval between the emission and excitation wavelengths (Δλ) at 15 nm or 60 nm, the properties of Tyr or Trp residues can be effectively characterized [37]. Changes in the microenvironment surrounding amino acid residues are reflected by shifts in the maximum emission wavelength. The maximum emission wavelength of HSA changes toward higher values (red shift) or lower values (blue shift), showing greater polar or hydrophobicity of the characteristic residues of Tyr or Trp, respectively. Figure 7 displays the effect of LUR on HSA on synchronous fluorescence spectra at Δλ = 15 nm and Δλ = 60 nm, respectively.

The presence of LUR caused the diminishment of emission spectra intensity (Figure 7), and this reduction at Δλ = 60 nm was considerably greater than that at Δλ = 15 nm. When Δλ = 15 nm, the presence of LUR did not cause changes in maximum emission wavelength, and consequently that of the Tyr residue did not change. By setting Δλ = 60 nm, we observed a small blue shift (about 2 nm) in the maximum emission wavelengths as well as a larger decrease in fluorescence intensity. This implies that LUR interacts with HSA through binding with a fluorophore closer to Trp, causing increasing the insignificance of non-polarity surrounding characteristic amino acid residues [38].

### 2.5. Molecular Docking

**Figure 8:**
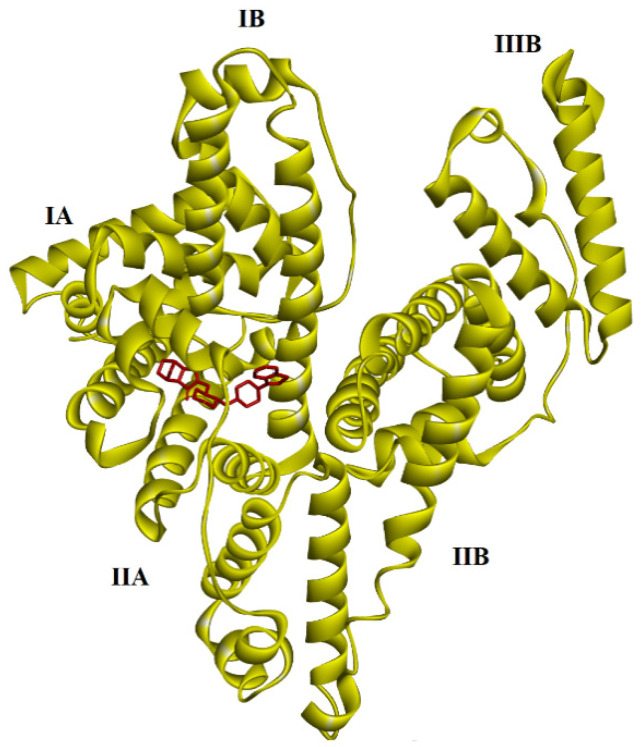
The results of blind docking between LUR and HSA proteins. LUR is marked by a red color.

The results of the theoretical calculations of examined complexes are displayed in Table 3. (with obtained ΔG and K_i_ values). First, blind docking was performed (in Table 3 represents the conformer of the lowest energy). The values obtained are as follows for favored site I subdomain IIA (Figure 8): ΔG = −9.91 kcal × mol^−1^; −41.46 kJ mol^−1^. The inhibition constant is 5.40 × 10^−6^ (M^−1^).

**Figure 9:**
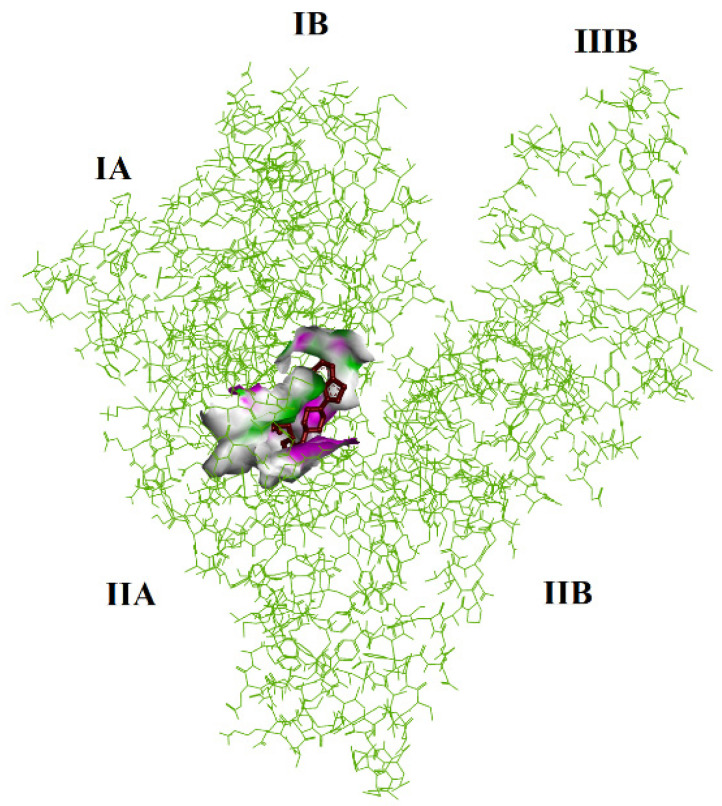
The binding affinity of LUR and HSA protein. LUR is marked by a red color.

Therefore, we focused on the favored side by repeating the calculations. There is a negative value in the case of the LUR (−8.17 kcal × mol^−1^; −34.18 kJ mol^−1^) complex. The inhibition constant is 1.02 × 10^−6^ (M^−1^). Thus, the represented data in Table 3 predict the strong binding of LUR to HSA at site I subdomain IIA (Figure 9).

**Figure 10:**
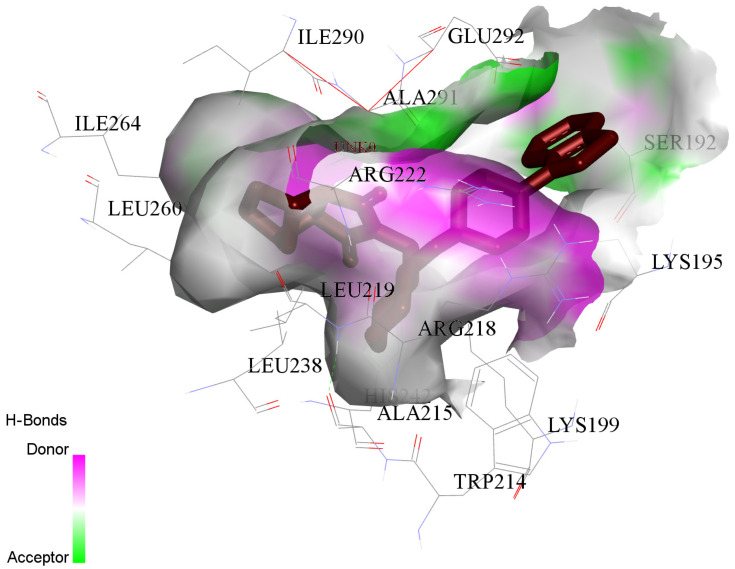
The principal contributions of the interaction between LUR and HSA proteins. LUR is marked by a red color.

The principal attraction between LUR and HSA comes from a conventional hydrogen bond (HIS A:242), van der Waals (LYS A:199; ARG A:222; ALA A:291), Pi-Sigma (LYS A:195), and Alkyl and Pi-Alkyl (TRP A:214; ALA A:215; ARG A:218; LEU A:238; LEU A:219; ILE A:264; ILE A:290; LEU A:260), as shown in (Figure 10).

## 3. Materials and Methods

### 3.1. The Materials

Fatty acid-free human serum albumin (HSA, A1887), lurasidone hydrochloride (LUR, 367514-88-3), phosphate-buffered saline (PBS, P4417), warfarin (WF, A2250), and ibuprofen (IP, I4883) were purchased from the Sigma–Aldrich Chemical Company (St. Louis, MO, USA) and used without further purification. The deionized water was used to prepare all solutions.

### 3.2. Preparation of Protein and Ligand Solutions

The phosphate-buffered saline (0.01 M PBS, 0.0027 M KCI, 0.137 M NaCl, pH = 7.4 at 298 K) was prepared by dissolving one tablet in 200 mL of deionized water. The stock solution of HSA (2 × 10^−5^ M) was prepared in a PBS solution. The stock solution of LUR was prepared by dissolving it in double-distilled water, and the sample was then diluted to 4 × 10^−5^ M with a solution of PBS. The WF and IP were dissolved in 5% ethanol and the sample was diluted to 4 × 10^−5^ M with a PBS solution. Until the experiments were performed, all solutions were stored in a cold (0–4 °C) and dark place.

### 3.3. Ligand Binding Studies

The interactions of lurasidone with HSA were studied using fluorescence spectroscopy [39,40]. The concentration of HSA was maintained at 1.6 × 10^−6^ M, while the ligand concentrations ranged from 0 to 3.2 × 10^−6^ M. The samples were allowed to equilibrate for 30 min before fluorescence measurements. The fluorescence spectra of these samples were recorded at three temperatures (298, 303, and 308 K) on an RF-6000 spectrofluorometer (Shimadzu, Kyoto, Japan) equipped with a thermostat bath, using a 150 W Xenon lamp source, 1.0 cm quartz cells, and a thermostatic bath. The samples were excited at 295 nm, and the emission spectra were recorded in the wavelength range of 300–450 nm. All fluorescence measurements were performed in three independent repetitions, and the results are expressed as mean values ± standard deviation.

### 3.4. Competitive Ligand Binding Studies

To determine the binding sites of LUR on HSA, competitive experiments were carried out using site-specific markers. The experiments were performed in the presence of fixed equimolar concentration (1.6 × 10^−6^ M) of ibuprofen (IP, site marker II)/warfarin (WF, site marker I) and HSA at different concentrations (0 to 3.2 × 10^−6^ M) of LUR. The fluorescence spectra were recorded at 296 K.

### 3.5. Analysis of the Binding Data

The fluorescence intensity was corrected for the absorption/reabsorption of excited light/of the emitted light according to Equation (1):

(1) $$F_{cor}=F_{obs}\text{×}10^{\frac{A_{ex}+A_{em}}{2}}$$

where *F*_cor_ and *F*_obs_ are the fluorescence intensities, corrected and observed, and *A*_ex_ and *A*_em_ are the absorptions of the system f excitation and emission wavelengths, respectively.

To reveal the quenching mechanism of HSA fluorescence, the Stern–Volmer equation was used [39] (2):

(2) $$\frac{F_{0}}{F}=1+K_{q}\tau_{0}\left[Q\right]=1+K_{\mathrm{SV}}\left[Q\right]$$

where *F*_0_ and *F* are the emission intensities in the absence or the presence of the quencher, respectively, *K*_SV_ is the Stern–Volmer quenching constant, *K*_q_ is the bimolecular quenching rate constant, *τ*_0_ is the average lifetime of the biomolecule without quench and it is equal to 8.0 ns for HSA [41] (*τ*_0_ = 10^8^ s), and [Q] is the concentration of the quencher. The binding constant (*K*_a_) and the number of binding sites of HSA (*n*) were calculated using the modified double-logarithm regression equation [40,42] (3):

(3) $$\log\frac{F_{0}-F}{F}=\log K_{a}+n\log\left(\left[Q\right]-[P](F_{0}-F)/F_{0}\right)$$

where [Q] and [P] are the total concentrations of the quencher and HSA, respectively. A plot of log(F_0_ − F)/F versus log([Q] − [P](F_0_ − F)/F_0_) gives a straight line that intercepts the *Y*-axis and its slope represents *K*_a_ and n, respectively.

The fluorescence measurements conducted at various temperatures (298, 303, and 308 K) enabled the calculation of thermodynamic parameters and facilitated the determination of the drug’s binding mode to macromolecules [27]. The thermodynamic parameters, including the enthalpy change (Δ*H*^0^) and entropy change (Δ*S*^0^), were derived using the van’t Hoff Equation (4) as follows:

(4) $$\ln K_{a}=-\frac{\Delta H^{0}}{RT}+\frac{\Delta S^{0}}{R}$$

where *Ka* is the binding constant, *R* is the gas constant, and *T* is temperature. The values of Δ*H*^0^ and Δ*S*^0^ were obtained from the slope and y-intercept of the van’t Hoff plot, respectively.

Equation (5) was used for the determination of the Gibbs free energy change (Δ*G*^0^):

(5) $$\left.\Delta G^{0}=-RT\;\ln\;K_{a}=\Delta H^{0}-T\Delta S^{0}\right.$$

### 3.6. Ultra-Violet Spectroscopy

A UV-vis absorption Perkin Elmer UV/Vis Lambda 365 spectrophotometer (PerkinElmer, Waltham, MA, USA), paired with a Xenon lamp and 1 cm path length cell, was exploited to record the UV-vis absorption spectra. The all measurements were performed in the range of 240–340 nm at room temperature. The concentration of HSA was fixed at 1.6 × 10^−6^ M, with the LUR varying from 0 to 3.2 × 10^−6^ M.

### 3.7. Circular Dichroism (CD) Spectral Measurement

The CD spectra were recorded using a JASCO J-815 (JASCO, Hachioji, Japan) spectropolarimeter equipped with cells of 1 mm path length. The scan speed and bandwidth were set at 100 nm/min and 2 nm, respectively. The scans were conducted at room temperature, maintaining a specific HSA concentration (2 × 10^−6^ M), and the molar ratios of HSA to LUR were kept at 1:0 and 1:3.5.

### 3.8. Synchronous Fluorescence Spectra

All spectra were measured at 298 K by setting Δλ = 15 and 60 nm for the aromatic amino acid residues of tyrosine and tryptophan, respectively. For synchronous fluorescence spectral measurements, the scanning interval (Δλ, λem − λex) was set at 15 nm to analyze changes in the microenvironment around Tyr residues and at 60 nm to assess alterations related to Trp residues, indicating the binding effects of LUR [37]. All spectra were measured at 298 K.

### 3.9. Molecular Docking

Lurasidone was downloaded from the PubChem Compound database (https://pubchem.ncbi.nlm.nih.gov/compound/213046, accessed on 12 February 2025). The available protein 3D structure of HSA (PDB code 1HK1) was acquired from the Protein Data Bank (PDB) (RCSB PDB: Homepage). Docking processes were carried out using Autodock 4.2 software equipped with the graphical user interface (GUI) Auto-DockTools (ADT 1.5.6rc3) [43,44]. The analysis of the LUR-HSA system model was based on the hydrogen bonds’ interactions. The outcomes of free binding energy and inhibition constants were acquired by ADT computational simulations. For the visualization of the docking results, a free version of the Discovery Studio Visualizer 3.5.0 Accelrys Software Inc. was used [45].

## 4. Conclusions

This study comprehensively explores the binding features between LUR and HSA. The decrease in HSA fluorescence intensity with increasing LUR concentration indicates complex formation. The stability of the HSA-LUR complex decreases with an increasing temperature, indicating a static quenching mechanism. Thermodynamic parameters reveal an endothermic, disordering interaction dominated by hydrophobic forces, with a single binding site for LUR on HSA. Site-selective binding experiments confirm LUR’s predominance in site I, specifically subdomain IIA, while conformational changes in HSA suggest partial chain unfolding due to LUR’s insertion into the hydrophobic cavity. These findings enhance our theoretical insights into LUR’s behavior, binding mechanisms, and pharmacokinetics, providing a foundation for future in vivo studies that can better elucidate its transport, distribution, and potential therapeutic effects. This study represents the first detailed exploration of LUR–HSA interactions at the molecular level, using a synergistic experimental and computational approach, thereby filling a critical knowledge gap in understanding how LUR interacts with key plasma proteins. The significance of these results lies in their contribution to the broader understanding of the molecular interactions between LUR and HSA with potential applications in drug design and optimization and in predicting the drug’s behavior within biological systems. LUR represents an important option in the treatment of schizophrenia and bipolar disorder, providing practical benefits and positive ripple effects in patient management and healthcare resource utilization, and its clinical success encourages the development of new antipsychotic medications with similar or improved profiles.

## Figures and Tables

**Table 1:** Quenching constants and rate constants for the interaction between HSA and LUR at different temperatures.

System	T (K)	*K*_SV_ × 10^5^ (M^−1^)	*Kq* × 10^13^ (M^−1^ s^−1^)	R^2 a^
HSA-LUR	298	2.51 ± 0.04	2.51	0.986
HSA-LUR	303	2.43 ± 0.04	2.43	0.982
HSA-LUR	308	2.30 ± 0.02	2.30	0.998

^a^ R is the correlation coefficient for Stern–Volmer plots.

**Table 2:** Binding constants (*Ka*), the number of binding sites (*n*), and the thermodynamic parameters of the interaction between HSA and LUR at different temperatures.

*T* (K)	*K*_a_ × 10^6^ (M^−1^)	*n*	R^2^	Δ*H*^0^ (kJmol^−1^)	Δ*S*^0^ (JK^−1^mol^−1^)	Δ*G*^0^ (kJmol^−1^)
298	3.98 ± 0.06	1.22	0.993			−37.71
303	8.13 ± 0.07	1.28	0.992	95.97	448.58	−39.95
308	13.99 ± 0.07	1.32	0.992			−42.19

R is the correlation coefficient.

**Table 3:** Results of molecular docking of LUR with HSA.

System	T ^a^	Δ*G* ^b^	Δ*G* ^c^	*K_i_*^d^ 10^−6^	Site (Subdomain)
Lurasidone (blind) *	298	−9.91	−41.46	5.40	I/IIA
Lurasidone	298	−8.17	−34.18	1.02	I/IIA

^a^ K; ^b^ kcal × mol^−1^; ^c^ kJ × mol^−1^; ^d^ M^−1^,* blind = blind docking.

## Data Availability

The data presented in this study are available upon request from the corresponding author.

## References

[1] Du X., Li Y., Xia Y.L., Ai S.M., Liang J., Sang P., Ji X.L., Liu S.Q. (2016). Insights into Protein–Ligand Interactions: Mechanisms, Models, and Methods. Int. J. Mol. Sci..

[2] Siddiqui S., Ameen F., ur Rehman S., Sarwar T., Tabish M. (2021). Studying the interaction of drug/ligand with serum albumin. J. Mol. Liq..

[3] Spada A., Emami J., Tuszynski J.A., Lavasanifar A. (2021). The uniqueness of albumin as a carrier in nanodrug delivery. Mol. Pharm..

[4] Jin M., Zhu S., Hou Y. (2023). Insight on serum albumin: From structure and biological properties to functional biomaterials for bone repair. ACS Biomater. Sci. Eng..

[5] Park J., Kim M.S., Park T., Kim Y.H., Shin D.H. (2021). Crystal structure of pharmaceutical-grade human serum albumin. Int. J. Biol. Macromol..

[6] Zsila F. (2013). Subdomain IB is the third major drug binding region of human serum albumin: Toward the three-sites model. Mol. Pharm..

[7] Radomska K., Wolszczak M. (2022). Spontaneous and ionizing radiation-induced aggregation of human serum albumin: Dityrosine as a fluorescent probe. Int. J. Mol. Sci..

[8] Ayimbila F., Tantimongcolwat T., Ruankham W., Pingaew R., Prachayasittikul V., Worachartcheewan A., Prachayasittikul V., Prachayasittikul S., Phopin K. (2024). Insight into the binding mechanisms of fluorinated 2-aminothiazole sulfonamide and human serum albumin: Spectroscopic and in silico approaches. Int. J. Biol. Macromol..

[9] Heng W., Su M., Cheng H., Shen P., Liang S., Zhang L., Qian S. (2019). Molecular interactions of drugs. Mol. Pharm..

[10] Guilera T., Pascual J.P.C., del Carmen Blasco M., Estopiñán P.C., González R.A.P., Martínez I.R., Moyano C.R., Pérez R.P., Gabarda-Inat I., Prados-Ojeda J.L. (2023). Lurasidone for the treatment of schizophrenia in adult and paediatric populations. Drugs Context..

[11] Ceskova E. (2022). Critical appraisal of lurasidone in the management of schizophrenia. Expert. Opin. Pharmacother..

[12] Stahl S.M., Morrissette D.A., Ritsner M.S. (2013). Should high dose or very long-term antipsychotic monotherapy be considered before antipsychotic polypharmacy?. Polypharmacy in Psychiatry Practice: Multiple Medication Use Strategies.

[13] Caccia S., Pasina L., Nobili A. (2012). Pharmacokinetics of antipsychotics. Neuropsychiatr. Dis. Treat..

[14] Danek P.J., Wójcikowski J., Daniel W.A. (2020). The atypical neuroleptics iloperidone and lurasidone inhibit human cytochrome P450 enzymes in vitro. Evaluation of potential metabolic interactions. Pharmacol. Rep..

[15] Starosta R., Santos F.C., de Almeida R.F. (2020). Human and bovine serum albumin time-resolved fluorescence: Tryptophan and tyrosine contributions, effect of DMSO and rotational diffusion. J. Mol. Struct..

[16] Hong Y., Feng C., Yu Y., Liu J., Lam J.W.Y., Luo K.Q., Tang B.Z. (2010). Quantitation, visualization, and monitoring of conformational transitions of human serum albumin by a tetraphenylethene derivative with aggregation-induced emission characteristics. Anal. Chem..

[17] Crouse H.F., Petrunak E.M., Donovan A.M., Merkle A.C., Swartz B.L., Basu S. (2011). Static and dynamic quenching of tryptophan fluorescence in various proteins by a chromium (III) complex. Spectrosc. Lett..

[18] Lakowicz J.R., Weber G. (1973). Quenching of protein fluorescence by oxygen. Detection of structural fluctuations in proteins on the nanosecond time scale. Biochemistry.

[19] Yang F., Zhang Y., Liang H. (2014). Interactive association of drugs binding to human serum albumin. Int. J. Mol. Sci..

[20] Tian F.F., Li J.H., Jiang F.L., Han X.L., Xiang C., Ge Y.S., Liu Y. (2012). The adsorption of an anticancer hydrazone by protein: An unusual static quenching mechanism. RSC. Adv..

[21] Feroz S.R., Mohamad S.B., Bujang N., Malek S.N.A., Tayyab S.J. (2012). Multispectroscopic and Molecular Modeling Approach to Investigate the Interaction of Flavokawain B with Human Serum Albumin. J. Agric. Food Chem..

[22] Berić J.D., Stojanović S.D., Mrkalić E.M., Matović Z.D., Milovanović D.R., Sovrlić M.M., Jelić R.M. (2018). Interaction of haloperidol with human serum albumin and effect of metal ions on the binding. Monatshefte für Chemie.

[23] da Silva Fragoso V.M., de Morais Coura C.P., Hoppe L.Y., Soares M.A.G., Silva D., Cortez C.M. (2016). Binding of sulpiride to seric albumins. Int. J. Mol. Sci..

[24] Huang Z.Y., Li X.Y., Hu L.Y., Bai A.M., Hu Y.J. (2022). Comparative study of two antipsychotic drugs binding to human serum albumin: By multispectroscopic and molecular docking methods. J. Mol. Liq..

[25] Caraci F., Sultana J., Drago F., Spina E. (2017). Clinically relevant drug interactions with anti-Alzheimer’s drugs. CNS Neurol. Disord. Drug. Targets..

[26] Sengul M.C.B., Karadag F., Sengul C., Karakulah K., Kalkanci O., Herken H. (2014). Risk of psychotropic drug interactions in real world settings: A pilot study in patients with schizophrenia and schizoaffective disorder. Klin. Psikofarmakol. B..

[27] Ross P.D., Subramanian S. (1981). Thermodynamics of Protein Association Reactions: Forces Contributing to Stability. Biochemistry.

[28] Rezaei-Tavirani M., Moghaddamnia S.H., Ranjbar B., Amani M., Marashi S.A. (2006). Conformational study of human serum albumin in pre-denaturation temperatures by differential scanning calorimetry, circular dichroism and UV spectroscopy. BMB Rep..

[29] Bertucci C., Domenici E. (2002). Reversible and covalent binding of drugs to human serum albumin: Methodological approaches and physiological relevance. Curr. Med. Chem..

[30] Ghuman J., Zunszain P.A., Petitpas I., Bhattacharya A.A., Otagiri M. (2005). Structural basis of the drug-binding specificity of human serum albumin. J. Mol. Biol..

[31] Sharma A.S., Anandakumar S., Ilanchelian M. (2014). A combined spectroscopic and molecular docking study on site selective binding interaction of Toluidine blue O with Human and Bovine serum albumins. J. Lumin..

[32] Abdelhameed A.S., Alanazi A.M., Bakheit A.H., Hassan E.S., Herqash R.N., Almutairi F.M. (2019). Novel BTK inhibitor acalabrutinib (ACP-196) tightly binds to site I of the human serum albumin as observed by spectroscopic and computational studies. Int. J. Biol. Macromol..

[33] Jafari-Arvari H., Saei-Dehkordi S., Farhadian S. (2021). Evaluation of interactions between food colorant, tartrazine, and Apo-transferrin using spectroscopic analysis and docking simulation. J. Mol. Liq..

[34] Nazzaro A., Lu B., Sawyer N., Watkins A.M., Arora P.S. (2023). Macrocyclic β-Sheets Stabilized by Hydrogen Bond Surrogates. Angew. Angew. Chem. Int. Ed..

[35] Zhao L., Zhang J., Zhang Y., Ye S., Zhang G., Chen X., Jiang J. (2021). Accurate machine learning prediction of protein circular dichroism spectra with embedded density descriptors. JACS. Au.

[36] Micsonai A., Moussong É., Wien F., Boros E., Vadászi H., Murvai N., Lee Y.-H., Molnár T., Réfrégiers M., Goto Y. (2022). BeStSel: Webserver for secondary structure and fold prediction for protein CD spectroscopy. Nucleic Acids Res..

[37] Amézqueta S., Beltrán J.L., Bolioli A.M., Campos-Vicens L., Luque F.J., Ràfols C. (2021). Evaluation of the interactions between human serum albumin (HSA) and non-steroidal anti-inflammatory (NSAIDs) drugs by multiwavelength molecular fluorescence, structural and computational analysis. Pharmaceuticals.

[38] Vesović M., Jelić R., Nikolić M., Nedeljković N., Živanović A., Bukonjić A., Mrkalić E., Radić G., Ratkovićc Z., Kljun J. (2024). Investigation of the interaction between S-isoalkyl derivatives of the thiosalicylic acid and human serum albumin. J. Biomol. Struct. Dyn..

[39] Lakowicz J.R. (2006). Principles of Fluorescence Spectroscopy.

[40] Mrkalić E., Jelić R., Stojanović S., Sovrlić M. (2021). Interaction between olanzapine and human serum albumin and effect of metal ions, caffeine and flavonoids on the binding: A spectroscopic study. Spectrochim. Acta A Mol. Biomol. Spectrosc..

[41] Liu Y., Li Q.Y., Wang Y.P., Liu Y.M., Liu B., Liu M.M., Liu B.M. (2018). Spectroscopic investigation of the anticancer alkaloid piperlongumine binding to human serum albumin from the viewpoint of drug delivery. Luminescence.

[42] Bi S., Song D., Tian Y., Zhou X., Liu Z., Zhang H. (2005). Molecular spectroscopic study on the interaction of tetracyclines with serum albumins. Spectrochim. Acta Part A Mol. Biomol. Spectrosc..

[43] Dassault Systèmes BIOVIA (2016). Discovery Studio.

[44] Morris G.M., Huey R., Lindstrom W., Sanner M.F., Below R.K., Goodsell D.S., Olson A.J. (2009). AutoDock4 and AutoDockTools4: Automated docking with selective re-ceptor flexibility. J. Comput. Chem..

[45] Sanner M.F. (1999). Phyton: A programming language for software integration and development. J. Mol. Graph. Model..

